# What Are We Here For?

**Published:** 1886-07

**Authors:** 


### "WHAT ARE WE HERE FOR?"

This celebrated interrogatory of the immortal Web Flanagan has been answered by the President of "*The*" Association of American What'd-ye-call-'em Physicians, thus:

"We all of us know why we are assembled here to-day. It is because we want an association in which there will be no medical politics and no medical ethics." (Francis Delafield, President of "THE" Association of American Physicians.)

Exactly. These self-righteous Philadelphia, Boston and New York doctors who have set themselves up as embodying *all* the brains, *all* the science, *all* the respectability of American medicine, want an association where they can run things in their own interest;—one which, having "no ethics," will take no cognizance of crookedness in the way of consultations with irregulars, and autographic newspaper puffs of their surgical operations. These Publican Specialists who no doubt "thank God that they are not as other men are"—(being considerably more "toney"), baffled in an attempt to "run the machine" in the A. M. A., set up opposition, and publish to the world that "this is the only genuine and great American article—all others are spurious—get the best."

We agree with the *Journal of the American Medical Association* that such an "Immaculate Conception" (and execution) should have some more distinctive title,—some trade mark to distinguish it from all other associations of "American Physicians;" something indicative and suggestive of the superior quality of the stuff of which it is composed—if not of the manner in which it is composed;—and we timidly venture as appropriate,—"The Association of The Few and Only (*soi-disant*) Pre-eminently Respectable and Scientific Practitioners of (Mixed) Medicine, Limited—(none genuine without the signature of *Osler Minot and Fitz*);" and their motto should be, "American Medicine in a Nutshell."

By the bye, will somebody kindly inform us who these gentlemen are? Who is Dr. Reginald H. Fitz—for instance? and what has *he* done?—and Dr. "Edes"—and "Dr. Osler?"—we never heard of either of those names until we learned that their owners had selected themselves as constituting each, the 100*th* of the sum total of American wisdom and science. We are ashamed to see Dr. Whitaker's name in this pie—("four and twenty black-birds all in a pie"); he belongs to the West, and was doubtless intended as a small "sop" to the Western Cerberus.

We believe conceit has not heretofore been recognized among the virtues, nor been considered an element in scientific attainment. As it is evidently a predominant feature in this organization, it must hereafter be recognized as both; and the rising generation should cultivate cheek, along with medical science, if they would become "distinguished American physicians."

